# Patterns of triggers, ideation and motivational factors of contraceptive utilization among women and gate-keepers in Nigeria: a scoping study on the resilient and accelerated scale up of DMPA-SC in Nigeria (RASUDIN)

**DOI:** 10.1186/s40834-020-00141-6

**Published:** 2020-12-01

**Authors:** Kehinde Osinowo, Michael Ekholuenetale, Oluwaseun Ojomo, Abiodun Hassan, Oladapo Alabi Ladipo

**Affiliations:** 1 Association for Reproductive and Family Health, 1st Floor, Block C, Millennium Builders Plaza, Abuja, Nigeria; 2 Department of Epidemiology and Medical Statistics, Faculty of Public Health, College of Medicine, University of Ibadan, Ibadan, Nigeria; 3 Family Health International (FHI360), Godab Plaza, J. S. Tarkar St, Garki, Abuja, Nigeria

**Keywords:** Family planning, women’s autonomy, Enabling factors, Parity, Pregnancy

## Abstract

**Background:**

Women have unfair share in the burden of unintended pregnancy outcome and unhealthy interpregnancy intervals. An understanding of the triggers, ideation and motivational factors influencing utilization of modern contraceptives is relevant for efforts aimed at increasing utilization among the general public, specifically sexually active women. The objective of this study is to explore the triggers, ideation and motivational factors influencing the use of modern family planning methods including depot-medroxyprogesterone acetate subcutaneous (DMPA-SC).

**Methods:**

Qualitative methods which include; Focus Group Discussions (FGDs) and In-depth Interviews (IDIs) were used to elicit information from women of reproductive age and gate-keepers in selected Nigerian states; Rivers, Ogun, Kwara, Niger, Anambra, Delta, Lagos, Enugu and Oyo. The categories of respondents include; unmarried women aged 18-25 years, women in union aged 18-24 years using modern family planning (FP), women in union aged 25-49 years using modern FP, women in union aged 26-49 years non-users of modern FP, community leaders, health facility focal person, husbands of current users of modern FP, husbands of non-users of modern FP, religious leaders, state FP coordinators and women aged 18-49 years who currently use DMPA-SC. Maximum variation sampling techniques was used to enlist participants to participate in both FGDs and IDIs.

**Results:**

Respondents reported being motivated to use FP for reasons such as benefits of the method, economic situation, suitability of the methods, fear of unwanted pregnancy and its convenience. Further analysis showed that the unmarried respondents discussed more about fear of unwanted pregnancy and accessibility and affordability as a key motivator; while women in union discussed more on economic situations, encouragement from partners and benefits of FP when compared with the unmarried. In addition, respondents reported that their partners, health workers and friends influenced their decisions to use FP. Partners’ encouragement, personal experience, accessibility and availability, awareness of FP and its benefits; willingness to space children and costs were notable enablers of FP use. The triggers for FP use were; appointment cards, phone calls from health workers, reminders (text messages, phone alarms and partners’ support).

**Conclusion:**

Increasing utilization therefore requires a well-planned horizontal approach that considers all enabling factors influencing utilization including women’s empowerment. Family planning programmes that are client centered, address socio-cultural and gender norms and ensure access to contraceptive mix methods are recommended to improve utilization rate. This study recommends improved care-seeking behaviour through community-based awareness creation to address myths and misconceptions of family planning use, establishment of contraceptive delivery teams to prevent challenges of availability and accessibility, value clarification and tasks shifting among others to deal with the issue of inadequate family planning utilization.

## Background

In the past decades, Nigeria has become one of the fastest growing population in the world due to low contraceptive use [1]. The estimated population is over 170 million while more than 40,000 women in Nigeria die annually from childbirth and related complications from unplanned pregnancies [2, 3]. Globally, family planning is the major approach to population growth control and enhance the economic dividends of the country. A recent report confirmed majority of people living below poverty line in Nigeria [4]; and this brings to limelight the benefits of family planning and the need to provide the opportunity to decide freely the number, timing and spacing of childbirth. Notably, more children survive and thrive when their parents can efficiently plan for their future and cater for them. Family planning is the nexus to the development of any nation and has been observed to be the gateway intervention to achieving the third Sustainable Development Goal (SDG-3) [5, 6].

Contraceptive use remains a prominent strategy to reduce the country’s maternal and infant mortality rates and prevent the spread of communicable diseases such as Human Immunodeficiency Virus (HIV)/Acquired Immunodeficiency Syndrome (AIDS) [7, 8]. Only healthy mothers can produce healthy children as family planning makes it easier for the mother to regain her strength and health after any delivery, she is able to focus on her own personal advancement and actively participate in paid employment, which invariably positively contributes to the country’s economy. Moreover, evidence have shown that many families are doing better because of their decision to uptake family planning [9], they can easily invest on their children and improve their overall wellbeing. Family planning has been proven to be a major contributing factor to gender equality and women empowerment which stands as a key factor in reducing poverty, sustaining economic growth and national development [9].

Regrettably, many women, especially those living in rural communities face many hardships while trying to access family planning, which are fueled by economic, social and cultural barriers. Some women live in communities where there are widespread socio-cultural beliefs that discourage them from using family planning, while some are misinformed, others do not have the resources or means to services [10, 11]. There are many cases of women who may want to uptake family planning, but are unable to do so because of key influencer decision makers in her life such as her husband, mother or mother-in-law. Some of these people may have strong negative influence on the woman, which may make it difficult for her to access such services, especially in Northern Nigeria. According to the Nigeria Demographic and Health Survey (NDHS), in spite of the gloomy situation, about one-fifth of women who desired to use one form of contraceptive method, were not using any method. This unmet need has not reduced, in spite of the huge investment in family planning interventions [12]. The implication of this is that a lot of sexually active individuals, are not using any contraceptives to prevent unintended pregnancies.

In this study, we explore the patterns of triggers, ideation and motivational factors affecting uptake of family planning in selected states of Nigeria. One of the flagship projects of the Association for Reproductive and Family Health (ARFH), Nigeria. This study sought to review factors and triggers and the determinants of contraceptive use, with interest and focus on ideational variables through the views and perceptions that women and gate-keepers. These views and ideas reflect individuals’ knowledge, opinions, ideas and views. Notably, mass media and social interaction channels contribute to shaping people’s views and ideas about family planning [2, 11, 13, 14]. There has been different assertion suggesting that ideational changes in the way people think as a key factor in fertility decline, changing moral values and decision making.

In the context of Social and Behaviour Change (SBC), there has been a lot of contextual association between different factors influencing the uptake of family planning services. It is important to determine the variables to close up the gaps and barriers to inform the appropriate SBC strategies for family planning intervention design. Giving the low contraceptive prevalence rate in Nigeria [15, 16], it is important to determine factors associated with modern contraceptive use staggering at approximately 10% among currently married women [12], which is significantly lower than the regional prevalence.

Furthermore, we set out to identify or do a deeper dive of major factors of modern contraceptive method uptake; some of which include poor spousal communication and support, sociocultural and religious norms, desire for a large family, fear of side-effects, lack of knowledge of different contraceptive methods as well as distance barrier to health facility amongst others [17–19]. The exploration of the triggers, ideation and motivational factors associated with family planning becomes very important prior to SBC which the “Resilient and Accelerated Scale Up of Depot-medroxyprogesterone acetate subcutaneous in Nigeria (RASUDIN)” project is set to adopt. Contraceptive use is influenced by people’s beliefs, ideas and changing the motivational factors can change people’s behaviour using multiple communication channels to foster understanding about family planning, increase social approval, and improve accurate knowledge about contraceptive methods. This study explored the use of the baseline findings from community audit in a project titled “Resilient and accelerated scale up of DMPA-SC/self-injection in Nigeria (RASUDIN)”. The project is being conducted in Ten Nigerian states to expand the acceptance, utilization, availability and accessibility of DMPA-SC within a broader contraceptive method mix, and kick start the roll out of Self-injection among women of reproductive age.

## Methods

### Study design

In this study, we utilized qualitative methods such as Focus Group Discussion (FGDs) and In-depth Interviews (IDIs) to elicit information from respondents. The qualitative study component utilized a phenomenological approach to explore the triggers, ideation and motivational factors influencing the use of modern family planning including DMPA-SC methods. This design was useful in understanding and describing the experiences of users and non-users of modern family planning methods including DMPA-SC methods. The qualitative study was conducted in nine [9] project states. See Table 1 for the details.

**Table 1:** Study carried out in nine out of 10 RASUDIN project states

State	Local government areas
Rivers	Phalga; Eleme; Obio-Akpor
Ogun	Ifo, Ado-Odo; and Abeokuta South
Kwara	Offa; Moro; Ilorin West; and Ilorin North.
Niger	Chanchaga; Busso; and Wushishi
Anambra	Aguata; Anaocha; and Idenmili
Delta	Isoko North; Oshimili South; Aniocha North; Ethiope East; Patani; and Sapele
Lagos	Lagos Mainland; and Ikorodu
Enugu	Enugu South; Enugu East; Enugu North; Udi-Agwu; and Nkanu West
Oyo	Akinyele; Ibadan South; Ogbomosho North; and Oyo West.

### Data collection

Study guides were developed for data collection to address knowledge, attitude, perception and barriers to modern family planning. In addition, the guide covered motivations, enablers and promoters of modern family planning including DMPA-SC. The FGD guide was used to facilitate group discussion and elicit response from participants; the paper-based note taking template was also used to document all responses in addition to audio recording of every session. Furthermore, IDI guide and note taking tool were also used. One-on-one interviews were conducted with identified stakeholders and persons with experiences, relevant to the focus of the research. Digital recording devices were used during the FGD and IDI sessions to record all discussions and interviews. Audio recordings were complementary to the notes taken in other to ensure comprehensiveness of notes. Both FGDs and IDIs interview sessions were facilitated by trained research assistants and note takers. A total of 56 FGDs and 71 IDIs were conducted across the 9 states. Total number of study participants for both FGD and IDI was 590. Table 2 shows a matrix of specific audience groups for FGDs and IDIs. The duration for FGDs and IDIs were approximately 60 min.

**Table 2:** Matrix of specific audience groups for FGDs and IDIs

Interviews	RASUDIN project states								
	Anambra	Delta	Enugu	Kwara	Lagos	Niger	Ogun	Oyo	Rivers
Unmarried women aged 18-25 years	FGD = 3	NA	NA	NA	NA	FGD = 3	NA	NA	FGD = 3
Women in union aged 18-24 years using modern FP	FGD = 2	FGD = 3	FGD = 3	FGD = 3	NA	FGD = 2	FGD = 4	FGD = 3	FGD = 3
Women in union aged 25-49 years using modern FP	FGD = 3	FGD = 3	FGD = 3	FGD = 3	FGD = 1	FGD = 3	FGD = 2	FGD = 3	FGD = 3
Women in union aged 26-49 years non-user of modern FP	IDI = 2	IDI = 2	IDI = 2	IDI = 2	IDI = 1	IDI = 3	IDI = 1	IDI = 1	IDI = 2
Community leader	IDI = 1	IDI = 1	NA	IDI = 1	NA	IDI = 1	IDI = 2	IDI = 1	IDI = 1
Health facility focal person	IDI = 1	IDI = 1	IDI = 1	IDI = 1	IDI = 1	IDI = 1	IDI = 1	IDI = 1	IDI = 1
Husbands of current users of modern FP	IDI = 1	IDI = 1	IDI = 1	IDI = 1	IDI = 1	IDI = 1	IDI = 1	IDI = 1	IDI = 1
Husbands of non-users of modern FP	IDI = 1	IDI = 1	IDI = 1	IDI = 1	NA	IDI = 1	IDI = 2	NA	IDI = 1
Religious leader	IDI = 1	IDI = 1	IDI = 1	IDI = 1	NA	IDI = 1	NA	NA	IDI = 1
State FP coordinator	IDI = 1	IDI = 1	IDI = 1	IDI = 1	NA	IDI = 1	IDI = 1	IDI = 1	IDI = 1
DKT State team	NA	NA	NA	NA	IDI = 1	NA	NA	NA	NA
Women aged 18-49 years who use DMPA-SC	NA	IDI = 1	IDI = 1	IDI = 1	IDI = 1	IDI = 1	IDI = 1	NA	NA
**Total**	**FGD = 8** **IDI = 8**	**FGD = 6** **IDI = 9**	**FGD = 6** **IDI = 8**	**FGD = 6** **IDI = 9**	**FGD = 1** **IDI = 5**	**FGD = 8** **IDI = 10**	**FGD = 6** **IDI = 9**	**FGD = 6** **IDI = 5**	**FGD = 9** **IDI = 8**

*NA* Not Applicable, *FGD* Focus Group Discussion, *IDI* In-depth Interview

### Sampling approach

Stratified purposeful and maximum variation sampling techniques was used to enlist participants to participate in both FGDs and IDIs. Stratification allowed the team to study subgroups of interest which included women of reproductive age disaggregated into two groups: age 18–24; and 25-49 years; users of modern contraceptives and non-users; husbands of users and non-users; women who use DMPA-SC; community and religious leaders; and family planning focal persons at the state and facility levels. Maximum variations sampling allowed us to explore how norms and perceptions varies across different groups and locations and socio-demographic variables such as age groups and marital status. Recruitment of target group was supported by staff of State Ministry of Health and ARFH state-based staff who served as mobilizers. These mobilizers ensured the right participants were selected and ready to participate in the interviews/FGD. Communication with the respondents was both in English and local dialects. The local dialects were later transcribed by experts of local dialects.

### Ethical approval

Ethical approval was obtained from National Health Research Ethics Committee (NHREC) of Nigeria – Protocol approval number: NHREC/01/01/2007–17/10/2018. In addition, permission to conduct the survey was also obtained from individual State Ministry of Health. All women and gate-keepers were informed of the purpose of the study, and they were assured of the confidentiality of information obtained. No names or specific contact information were obtained from study participants. Written informed consents for both the discussions and the audiotaping were obtained from the participants. Where the consent cannot be obtained in writing, the non-written consent was formally documented and witnessed. They were informed that evidence generated from the study could inform policies made and change practices leading to the reduction of maternal mortality. They were also informed that they would incur no risk if they opt out of the interview at any time. Only those who agreed to participate in the fully explained study and gave consent were recruited for the study.

### Data management

Recordings from interviews and focus group discussions were transcribed verbatim by a team of research assistants. Recordings in Hausa, Igbo and Yoruba languages were translated by experienced research assistants who also conducted the interviews in local dialects. All transcripts were exported into Dedoose 8.0, a qualitative software and analyzed based on thematic approach to identify themes and patterns across research objective areas. Coding of transcripts was carried out by a team of researchers from ARFH and Centre for Communication and Social Impact (CCSI). The initial phase involved deductive coding where a set of predetermined codes reflecting main topical areas of the discussion guide applied. A harmonized coding structure was finalized by the research team who worked together to ensure consistent coding pattern, relevant themes were captured, and codes reflected the research objectives. Thereafter, inductive coding of transcripts was carried using themes and categorizing responses aimed at addressing the study objectives. Comparative assessments of patterns and themes were examined within themes and across sub-groups, and socio demographic variables used in the study; and emerging themes were identified and documented during the iterative coding process. Themes were identified with associated sub codes.

## Results

### Ideation and motivational factors

#### Motivations to use modern family planning (FP)

Concerning what motivates women to use family planning, it was observed that respondents were motivated to use FP for reasons such as benefits of the method, economic situation, and suitability of the methods, fear of unwanted pregnancy and its convenience. Further analysis showed that the unmarried respondents discussed more around fear of unwanted pregnancy and accessibility and affordability as a key motivator; while women in union discussed more around economic situations, encouragement from partners and benefits of FP when compared with the unmarried (See Table 3) below.

“*The condition of Nigeria influenced my decision to take up family planning*” **[FGD, women aged 18–24 years, Anambra].**

**Table 3:** Motivating factors for women to use modern family planning

Motivations	Number of coded segments
Fear of unwanted pregnancy	44
Benefits of FP	107
Economic situation	62
Affordability and accessibility	22
Personal conviction	33
Suitability and convenience	40
Referrals	8
Encouragement from partners	6
Others	31

“*Because if you born pass your level, who will feed them and take care of them, and the present state of the country …*” **[FGD, women in union 18-24 years, Enugu]**

“*The biting economy has made me use family planning*” **[FGD, women in union aged 18-24 years using modern FP]**

**Fig. 1:**
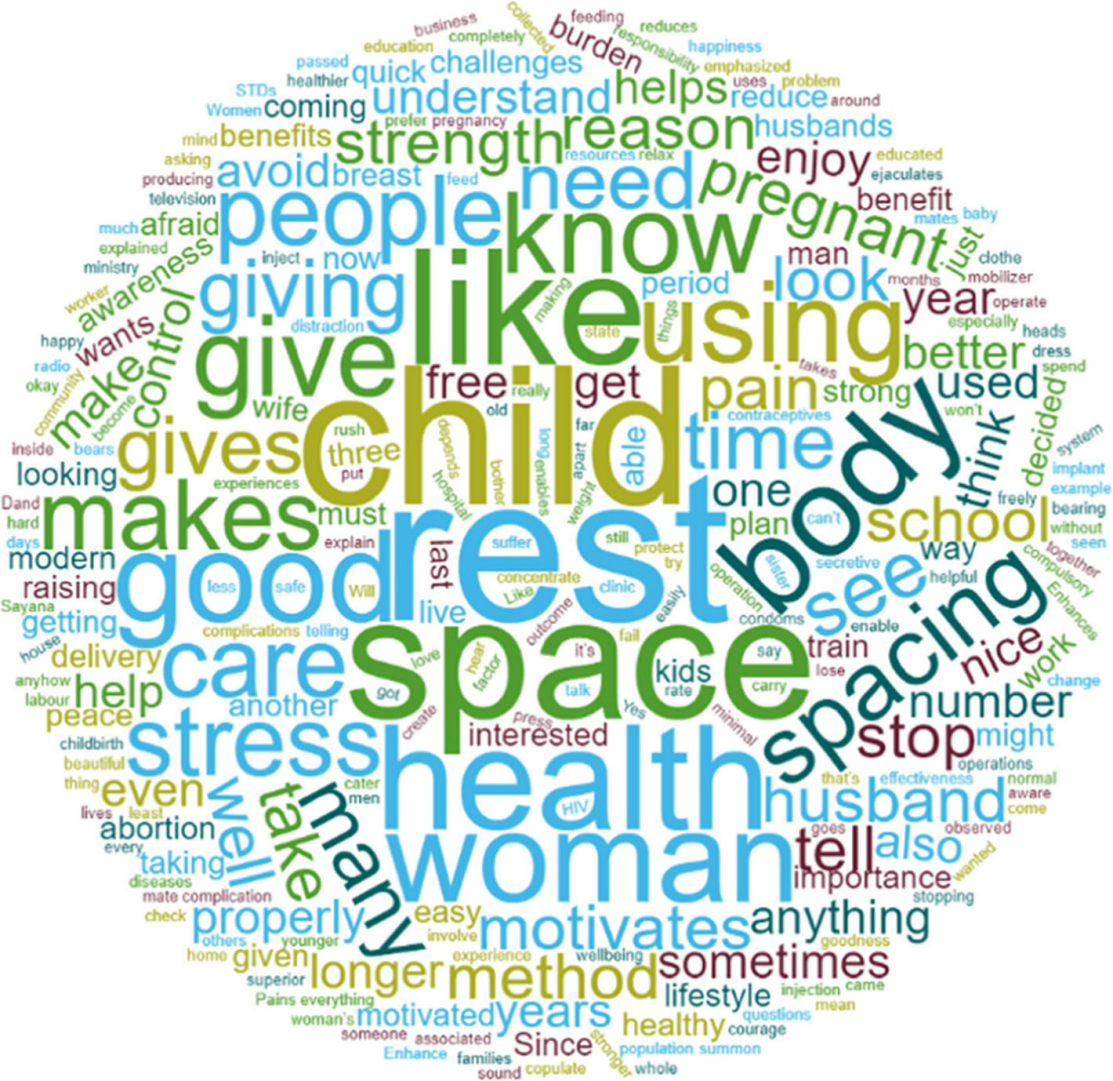
What respondents describe as benefits of FP

Further analysis to explore what respondents described as benefits of FP which motivates them include rest, good spacing leading to healthy mothers and children (Fig. 1, Table 4).

**Table 4:** Factors that encourage women to use modern FP

Encouraging factors	Number of coded segments
Affordability	21
Availability	26
Economic hardship	7
Experience of others	11
No side effects	43
Partners support	6
Sensitization/awareness	101
Others	35

#### Influencers of modern FP

More respondents reported that their partners influenced their decisions to use FP. Other influencers identified during the FGD/interview sessions include: health workers, self and friends.

This study revealed that the decision-making process regarding utilization of family planning services, is influenced by the decision maker in the family. Men are dominant in this process, but some women are also involved in making the decision as well. Excerpts from some respondents are stated as below (Table 5):

“*It is the couple but sometime the wife would sneak out and do it (family planning) if the man doesn’t care about her well-being*” **[FGD, women in union, 25–29 years, Enugu]**

“*Persistent request for sexual intercourse*” **[FGD, women aged 25-49 years, Oyo]**

**Table 5:** Decision makers regarding number of children

Decision makers	Number of coded segments
Both couple/partners	110
Husbands/Male partners	103
Wife/Female partners	58
Health providers	2
Mother-in-law	4
Religious leaders	2
Others	6

Another respondent from Anambra has this to say:

“*What made me to start using a family planning method was when I gave birth to my second daughter, she was like 7 months and I now took in again. It was too stressful with the pregnancy and I am going to work, taking care of the other child, school runs and the rest so it was not that easy, when I give birth to the third child, I and my doctor, we now discuss on the family planning because I asked the doctor if there is any way to avoid taking in so soon, so he now introduce the family planning to me. Initially I don’t like the idea but when he now explains the benefits and the rest and no side effects, I now admitted it, that was my experience*” **[FGD, women in union 18-24 years using modern FP, Anambra]**

Interestingly, most of the women were motivated to take up modern family planning method following previous history of personal negative experience of not using a method or a negative experience of someone close to them. Excerpts below describe how these women expressed these experiences in their own words:

“*Death of a neighbor when she was giving birth to the 10*^*th*^ *child*” **[FGD women in union aged 18-24 years, Rivers]**

**“***My girlfriend got pregnant 2 or three times and had abortions, that made me use family planning*” **[FGD Unmarried women aged 18-25 years, Rivers]**

“*… I met a woman who was poor, even to pay the children school fees was the problem but the husband will always want her to give birth. I ask her what was the problem, she said that the man said that he is the only son of his parent. I told her the best thing for you now is to quietly do FP method without the husband knowing, because the husband won’t even agree to it. Another example is a man who got married to a girl at her tender age and the man just want her to be giving birth and the girl was tired I told her is just to use modern FP quietly …”* **[IDI religious leader, Enugu]**

“*After giving birth to my first daughter, I was using withdrawal method and the method failed me and produced this baby, now I am using the pills and I am okay with it*” **[FGD, Women in union aged 18–24 using modern FP, Anambra]**

“*My neighbor who is above 40years of age got pregnant after 10 years. She thought she had stopped and the pregnancy came in when she didn’t expect it. That was what I saw that made me use modern FP method*” **[FGD, Women in union aged 18-24 years using modern FP, Ogun]**

“*I also use tablet method there was a particular month that I forgot to use it and I got pregnant, on the naming day my husband ran out from the house luckily for me I was working in the secretariat as a cleaner and I was also involve in thrift in the office that was how I use the money to do my naming ceremony when I explain to my friend she then advise that I use the implant method of 5 years which I did*” **[FGD, Women in union aged 25-49 years using modern FP, Ogun]**

“*The reason I decided to use FP is because of my daughter. I just gave birth to the previous pregnancy, I suffered so much I didn’t even know that I was going to be alive so now I want to rest at least 2-3years*” **[IDI, Women aged 18-49 years who use DMPA-SC self-inject, Niger]**

**“***My friend told me her mother uses Igi Ibepe (meaning fused-end of a pawpaw fruit) and after some time it failed, and she got pregnant after which she aborts the pregnancy, now she’s using modern FP, so, that experience made me use modern FP*” **[FGD, Women in Union aged 18-24 years using modern FP, Ogun]**

### Enablers, internal and external triggers

When asked what made it easy for partners to use FP, respondents mentioned the following common factors: Partner encouragement, personal experience, Accessibility and availability, awareness of FP and its benefits; willingness to space children and costs. Excerpts below describes some of the views of respondents:

“*It is my husband because he wants it, so after talking to me about it and giving me much advice on what I will gain from it, I have to obey and it favoured me also*”. **[FGD, women in union, 18–24 years, Enugu state].**

“*Some people believe if you are on family planning in your next world you will not have a child. Some people too, they believe that if you do family planning, you will be barren for life, even when you want to have a child, you won’t have a child*” **[IDI, FP focal person, Rivers]**

“*They don’t like it, some of them because I know of a man whose wife gave birth to triplet as her first issue and she is a young girl that just got married, I spoke with the husband to let her take family planning because it is not easy for her with the 3 children at once and she doesn’t have any helper, the man said no that if the wife knows about it, she will start misbehaving because she is free and if she does it, she cannot get pregnant so it will be an eye opener for the girl. She is young and still in secondary school, he said no, he doesn’t want her to go that side because if she does it will be an eye opener and she will know more than she should know*”. **[FGD, women in union, 25–49 years, Enugu]**

Similarly, respondents were asked what would make it easy for them to continue use of FP. More mentions were made of the following: use of appointment cards, phone calls from health workers, reminders (text messages, phone alarms and reminders from partners).

“*Some of these family planning last for 3 months, people like me it might even pass 6 months before I will remember that I am supposed to renew it every 3 months so it better for them to be sending alert, when you go to the nurse, she should register you and send you a reminder of the month you are to come back or else we will forget*” **[FGD Women in union aged 25-49 years using modern FP, Anambra]**

“*They can tell her husband to remind her about her appointment*” [**IDI, Husbands of current user, Niger]**

“*Where I did my own, they gave me a card and they wrote the date and the month when I will come back to renew it. It makes it easier for me to remember*” **[FGD, women in union aged 18-24 years using modern FP, Rivers]**

More women from Oyo and Niger states mentioned the use of appointment cards as an enabler for continued use of FP. Among Kwara respondents, there was more mention of phone calls and reminders (text messages, partner’s reminder).

The study also revealed that FP presents parents with the opportunity to properly train their children due to proper spacing (Table 6).

“*The health centre is near to my home, so it makes it easy for me and the charges is a little amount compare to the expenses you will spend when you get pregnant*” **[FGD, women in union 25–49 years, Enugu].**

“*Sincerely even if you have not decided to do FP, the way it is been advertised, publicized and the way its benefits are explained, you will want to do it like me it is injectable I use but the way I see people using the implants me too I will love to put it*”**[FGD, women in union aged 25-49 years, Niger].**

**Table 6:** Identified Motivators, Abilities and Triggers

Motivators	Abilities	Triggers
• Fear of unwanted pregnancy • Benefits of FP • Economic situation • Accessibility and Suitability • Encouragement from partners • Negative experiences from personal and close relatives • Long lasting methods • Methods with less side effects • Awareness about methods • Cost-Affordability • Influence from friends • Availability of methods • Effectiveness of methods • Privacy of methods	• Ability to self-administer • Willingness to use • Self will • Personal experience • Personal conviction	• Appointment cards • Phone calls • Text message reminders • Partner reminder • Home visits by health worker or community volunteer

## Discussion

In this study, we explored the triggers, ideation and motivational factors of modern family planning methods including DMPA-SC across selected states in Nigeria, including; Oyo, Ogun, Rivers, Enugu, Anambra, Delta, Niger, Kwara and Lagos. Though several factors were found to influence the utilization of modern contraceptive methods, there were disparities in the motivational and enabling factors that exerted high influence over utilization. Understanding of the benefits of contraceptive utilization, sensitization/awareness creation, lack of side effects, economic hardship and accessibility to contraceptive services; be it geographical or financial accessibility was found to exert considerable influence over contraceptive utilization. Based on the findings of this study, several other factors were reported to encourage or motivate women to use modern contraceptive methods. These include; fear of unwanted/unintended pregnancy, availability and affordability of contraceptive methods, suitability and convenience, recommendation by friends or health workers, encouragement from partners, confidentiality of contraceptive methods and effectiveness of contraceptive methods. Our findings suggest that an increase in contraceptive use can be achieved by enhancing women’s empowerment through spousal support, sensitization, awareness creation, financial support and positive peer influence. This brings to limelight, the effect of decision-making autonomy by women and improved knowledge level as major factors to be considered in health-related programmes to yield population control benefits to the societies in general [9].

Also, the cost of acquiring diverse contraceptive methods could determine the utilization rate. This implies that disadvantaged women will be most-at-risk of the consequences of not being able to afford contraceptives. These could include the cost to travel to a health facility or amount of time spent, or cost to purchase the service or commodities known as the indirect and direct cost respectively [20, 21]. Though family planning services in public health facilities may be with small fee for some methods, the costs of commodities could be high for some types of contraceptive methods. On the availability of contraceptive methods, though many of the health facilities and community clinics may claim to have varieties of modern contraceptives, only a limited number of key methods may actually be available. The most contraceptives methods that are usually available are condoms, injectables and pills. However, other methods like the DMPA-SC, intrauterine device (IUD), implant and other long term methods are less readily available or accessible. This finding is consistent with a previous report [11].

The fear of side effect for different contraceptive methods could hamper contraceptive use. To this effect, women may see it as a risky medical process which could lead to infertility, increase in weight gain and sometimes irregular bleeding. A study revealed that women who discontinued modern contraceptive methods explained that they found it difficult to give birth when they wanted to and that it took them long time to conceive, which was clearly unusual to them [22]. The perceptions of women about the side effects of modern contraceptives to a large extent also determine the level of utilization in Nigeria. Though most medicines and other medical procedures have their side effects, patients and clients continue to use them. However, with the use of modern contraceptive methods, side effects could be the major reasons why women could decline use. Common side effects which lead to non-use of contraceptive use have previously been reported to include waist and abdominal pains, high body temperature, increase in weight, irregular menstrual flow and hypertension had kept a number of clients away [23].

For the decision makers regarding the number of children to have in a family, prominently, respondents reported that both couple/partners and husband/male partner as key decision makers. Others such as wife/female partner, health workers, mother-in-law and religious leaders could also influence the family size. Some gender practices such as women’s inability to make decisions concerning their reproductive health, due to early and forced marriages could greatly influence women decision-making power on the number of children they wanted for the family. These poor marriage practices, specifically in several rural communities in Nigeria has taken away self-confidence of women and also put them in a position that makes it difficult to negotiate contraceptive use with their male partners when they would actually like to stop having children.

Notably, several abilities or enablers in contraceptive utilization identified in this study include ability to self-administer the chosen method, willingness to use, self-will, personal experience and conviction. Furthermore, the triggers identified for contraceptive use include; appointment cards, phone calls, text message reminders, partner’s reminder and home visits by health worker or community volunteers. The findings of this study suggest the need for interventions to promote healthy interpregnancy intervals to reduce adverse child health outcomes [24]. In addition, health care stakeholders would find the findings of this study interesting and a base for policy formulation and implementation.

## Conclusion

Increasing utilization therefore requires a well-planned horizontal approach that considers all enabling factors influencing utilization including women’s empowerment. Family planning programmes that are client centered, address socio-cultural and gender norms and ensure access to contraceptive mix methods is recommended to improve utilization rate. This study recommends improved care-seeking behaviour through community-based awareness creation to address myths and misconceptions of family planning use, establishment of contraceptive delivery teams to prevent challenges of availability and accessibility, value clarification and tasks shifting among others to deal with the issue of inadequate family planning utilization.

## Data Availability

Data is available strictly on request.

## Abbreviations

AIDS: Acquired Immunodeficiency Syndrome
ARFH: Association for Reproductive and Family Health
CCSI: Centre for Communication and Social Impact
DMPA-SC: Depot-medroxyprogesterone acetate subcutaneous
FGDs: Focus group discussions
FP: Family planning
HIV: Human Immunodeficiency Virus
IDIs: In-depth Interviews
IUD: Intrauterine device
NDHS: Nigeria Demographic and Health Survey
NHREC: National Health Research Ethics Committee
RASUDIN: Resilient and Accelerated Scale Up of Depot-medroxyprogesterone Acetate Subcutaneous in Nigeria
SBC: Social and Behaviour Change
SDG: Sustainable Development Goal
